# Characterisation of ATP analogues to cross-link and label P2X receptors

**DOI:** 10.1016/j.neuropharm.2008.05.018

**Published:** 2009-01

**Authors:** Kelvin C. Agboh, Andrew J. Powell, Richard J. Evans

**Affiliations:** a Department of Cell Physiology and Pharmacology, University of Leicester, P.O. Box 138, University Road, Leicester LE1 9HN, UK; b Biological Reagents and Assay Development, GlaxoSmithKline R&D, Stevenage, Herts SG1 2NY, UK

**Keywords:** ATP, P2X, Agonist, Binding, Cross-linking

## Abstract

P2X receptors are a distinct family of ATP-gated ion channels with a number of physiological roles ranging from smooth muscle contractility to the regulation of blood clotting. In this study we determined whether the UV light-reactive ATP analogues 2-azido ATP, ATP azidoanilide (ATP-AA) and 2′,3′-*O*-(4-benzoylbenzoyl)-ATP (BzATP) can be used to label the ATP binding site of P2X1 receptors. These analogues were agonists, and in patch clamp studies evoked inward currents from HEK293 cells stably expressing the P2X1 receptor. Following irradiation in the presence of these compounds subsequent responses to an EC_50_ concentration of ATP were reduced by >65%. These effects were partially reversed by co-application of ATP or suramin with the photo-reactive ATP analogue at the time of irradiation. In autoradiographic studies radiolabelled 2-azido [γ^32^P] ATP and ATP-AA-[γ^32^P] cross-linked to P2X1 receptors and this binding was reduced by co-incubation with ATP. These studies demonstrate that photo-reactive ATP analogues can be used to label P2X receptor and may prove useful in elucidating the ATP binding site at this novel class of ATP binding proteins.

## 1 Introduction

The physiological effects of ATP are mediated through the activation of ionotropic P2X receptors and G-protein coupled P2Y receptors (Burnstock, 2006; North, 2002). Seven P2X receptor subunits (P2X1–P2X7) have been identified and these associate together to form homo- or hetero-trimeric receptors with a range of phenotypes (North, 2002; Roberts et al., 2006). The P2X receptor membrane topology features two transmembrane-spanning segments, an extracellular loop and amino and carboxy termini which exist intracellularly. P2X receptors have been implicated in a number of physiological roles including regulation of blood clotting, smooth muscle contractility, as well as pain and accordingly P2X receptors are potential targets for pharmacological intervention (Roberts et al., 2006). The production of various pharmaceutical therapies by rational drug design has been hindered because the extracellular domain of P2X receptors contains a distinct ATP binding site that has yet to be fully elucidated. One of the complications is the lack of sequence homology with other ATP binding protein classes, i.e. none of the “classical” nucleotide binding motifs have been detected in P2X receptors. For example the motif GXXXXGKT/S (where X = any residue), more commonly known as the Walker motif A (Walker et al., 1982) is not present. Site-directed mutagenesis of conserved amino acid residues in the extracellular loop and the structural homology to other nucleotide binding proteins have been used to propose different models of the ATP binding site (Evans, in press; Yan et al., 2005). Although mutagenesis is a powerful tool in directing attention to specific residues that appear to influence ATP potency, discrimination between residues involved in agonist binding and residues involved in the process of gating is difficult.

Direct binding evidence can corroborate data from previous studies as well as indicate other residues which may play important roles in the ATP binding site. Radiolabelled ATP analogues such as 5′-(*p*-fluorosulfonyl)benzoyladenosine (FSBA) have been used to affinity label a range of ATP binding proteins (e.g. Figures et al., 1987). Similarly photo-reactive ATP analogues have been used to identify residues within the binding sites of many proteins including the Na^+^,K^+^-ATPase (Tran et al., 1994), K_ATP_ channels (Tanabe et al., 2000) and natriuretic peptide receptor (Joubert et al., 2005). The identification of a photo-reactive or cross-linking compound with activity at P2X receptors would be the first step in providing direct evidence of the identity of some of the amino acid residues which contribute to the ATP binding site, therefore we investigated whether FSBA, 2-azido ATP, ATP azidoanilide or 2′,3′-*O*-(4-benzoylbenzoyl)-ATP (BzATP) can be used to label the P2X receptor binding site.

## 2 Materials and methods

### 2.1 Cell culture

Native HEK293 and HEK293 cells which stably express the human P2X1 receptor or the human P2X4 receptor were cultured as previously described (Vial et al., 2004). Native HEK293 cells, at 80–90% confluence, were plated onto 40 mm cell culture dishes. For each dish of cells, transient transfection was conducted using 1 μg DNA (rat P2X2 and rat P2X3) and 10 μl lipofectamine 2000 (Invitrogen) in 500 μl of serum-free Opti-MEM1. To confirm the presence of transfected protein, GFP (green fluorescent protein) was co-transfected. After 6–10 h incubation, cells were plated onto 13 mm coverslips and left to grow in DMEM cell culture medium. Cells were subjected to experiments 24–48 h after transfection. Single cells that expressed green fluorescence implied expression of the transfected gene (P2X2 or P2X3) and were subjected to electrophysiological recording. The preparation of plates, cell culture and transfection all took place in a laminar flow hood under sterile conditions.

### 2.2 Electrophysiological recordings

Electrophysiological recordings were made using the whole cell patch clamp configuration on single cells at room temperature (21–26 °C). Microelectrodes were filled with internal solution composed (in mM) of K gluconate, 140; EGTA, 10; HEPES, 10; NaCl, 5 (pH 7.3, adjusted with KOH) and had resistances in the range of 2–6 MΩ. The bath was continuously perfused with extracellular solution containing (in mM) NaCl, 150; HEPES, 10; glucose, 11; KCl, 2.5; MgCl_2_, 1; CaCl_2_, 2.5 (pH 7.3, adjusted with NaOH). Cells were voltage clamped at −70 mV (after correction for the tip potential) and the data was low pass-filtered at 1 kHz and digitized at a sampling interval of 200 μs. Using an Axopatch 200B amplifier, data was collected with pClamp 8.1/9.0 software. Agonists were applied for 1 s via a fast-flow U-tube (Evans and Kennedy, 1994) and P2X1 receptor responses were quantified by measuring the peak current amplitude relative to the base-line holding current immediately preceding agonist application.

### 2.3 Cross-linking with photo-reactive ATP analogues

Wild-type P2X1 cells, mounted on 13 mm coverslips, were placed in an 8.8 cm^2^ petri dish (Nunclon, UK) and were incubated in the presence of 2 ml of 30 μM 2-azido ATP, 100 μM ATP azidoanilide (both Affinity Labelling Technologies Inc, USA) or 10 μM BzATP (Sigma, UK). A UV light source (Uvitech, UK, LF206.MS; Flowgen, Ashby Park, Leicestershire, UK) was housed in a box which was maintained at room temperature and constant humidity and excluded outside light. The cells were placed 7.5 cm from the UV light source and the wavelength was set at 312 nm. Cells were then continually irradiated for 3 min. After irradiation, cells were washed for 5 min in extracellular solution and were ready for electrophysiological recording.

### 2.4 Radiolabelled cross-linking

To detect the binding of 2-azido ATP or ATP azidoanilide to the P2X1 receptor, native HEK293 cells and wild-type P2X1 cells were cultured in 24-well plates in Dulbecco's modified Eagle's medium (DMEM) (Gibco) with Earle's salts, glutamax 1, 10% foetal calf serum and 1% non-essential amino acids. Wild-type P2X1 cells were maintained under permanent selection in 600 μg/ml G418 (Invitrogen). When cells reached 80% confluence the growth media was removed and replaced with either 1 μM 2-azido [γ^32^P] ATP (0.32 MBq) or 1 μM ATP-AA-[γ^32^P] (0.92 MBq) (both Affinity Labelling Technologies Inc, USA) at 0.5 ml/well. The 24-well plate containing these cells was placed in the UV box and continually irradiated by 312 nm of UV light for 3 min. The 24-well plate was placed on ice and unbound ^32^P labelled ATP analogue was removed by washing the cells twice with 1 ml PBS. The cells were then lysed into a homogenising buffer (100 μl) containing 150 mM NaCl, 1 mM EDTA, 1 mM EGTA, 40 mM Tris–HCl (pH7.4), 8 mM Tris–base, 1% triton X and a mixture of protease inhibitors (1:100 dilution) (Sigma). The cell homogenate was centrifuged for 5 min at 13,000 rpm (4 °C) on a benchtop centrifuge, the cell debris was discarded and the supernatant was stored on ice. A stock solution of P2X1 antibody (0.3 mg/ml) (Alomone, Israel) was prepared and 3 μl of the solution was added to 85 μl of the supernatant. The mixture was allowed to incubate on ice for 1 h. Protein A-sepharose beads (30 mg/ml) (Amersham, UK) were pre-washed in PBS and the resultant antibody–antigen complex was precipitated with the addition of 75 μl protein A-sepharose beads and rolled at 4 °C overnight. One millilitre of homogenising buffer was added to the protein A-sepharose beads (complexed with the antibody and antigen) and the mixture was spun on a benchtop centrifuge at 13,000 rpm for 5 min. The supernatant was removed with a pump aspirator and discarded. The process was repeated three times in total to enable the beads to be washed of any unbound radioactive material. Twenty microlitres of sample buffer (with 5% β-mercaptoethanol) were added to the protein A-sepharose P2X1 pellet. The pellet was heated to 95 °C for 5 min to allow the protein to elute from the beads. Twenty microlitres of the sample were removed and loaded onto a 10% SDS-PAGE gel and run at 120 V. For autoradiography, the gels were dried and exposed to X-ray film for 48–72 h at −80 °C.

### 2.5 Data analysis

The results are expressed as mean ± standard error of mean and analyzed using the appropriate Student's *t*-test. Data analysis was carried out with the computer package, “Excel” and the graphics package “Origin 6” was used to construct the graphs.

## 3 Results

### 3.1 Effects of the chemical cross-linking compound FSBA

The ATP analogue FSBA is a chemical cross-linking compound. FSBA (100 μM) had no agonist action at hP2X1 receptors stably expressed in HEK293 cells. Similarly following 1 h incubation with FSBA (a commonly used procedure to cross-link and block ATP binding sites) subsequent responses to 1 μM ATP (an ∼EC_50_ concentration) were no different to those recorded under control conditions (control peak amplitude −2.84 ± 0.28 and −2.76 ± 0.42 nA for control and following FSBA treatment, respectively, *n* = 10.6). These results show that FSBA is ineffective as an agonist or cross-linker at the P2X1 receptor.

### 3.2 2-Azido ATP is an agonist at human P2X1 receptors

**Fig. 1:**
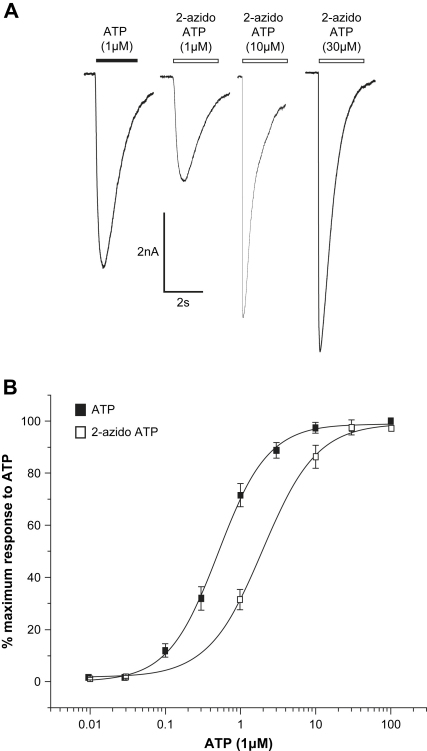
The agonist action of 2-azido ATP. (A) Whole cell patch clamp traces in response to agonists. ATP evoked a desensitizing, inward current (application of 1 μM ATP is indicated by black bar). The application of 2-azido ATP (indicated by the open bars) produced a similar desensitizing inward current that increased when the applied concentration of 2-azido ATP was increased. (B) Concentration–response curves to ATP and 2-azido ATP. Error bars indicate SEM.

2-Azido ATP has a reactive group substituted at the 2nd position on the adenine ring of ATP and may be useful for investigating amino acid residues close to the site of the adenine ring of ATP bound to P2X receptors. 2-Azido ATP evoked concentration dependent desensitizing inward currents at P2X1 receptors with an EC_50_ of ∼3 μM. This suggests that 2-azido ATP may be suitable for cross-linking to P2X receptors following UV illumination (Fig. 1).

### 3.3 The effects of UV irradiation of 2-azido ATP on P2X1 receptor currents

The photo-active azido group, upon UV irradiation, is converted into a highly reactive nitrene that can form covalent bonds that could be used to cross-link the compound to the P2X1 receptor. As a control, cells were UV irradiated for 3 min. They were then placed on the electrophysiology rig and application of 1 μM ATP elicited responses of −3.03 ± 0.57 nA (*n* = 6); this was not significantly different from untreated control cells (−3.47 ± 0.9 nA (*n* = 9)) and indicates that this UV irradiation protocol has no effect on the sensitivity of P2X1 receptors.

**Fig. 2:**
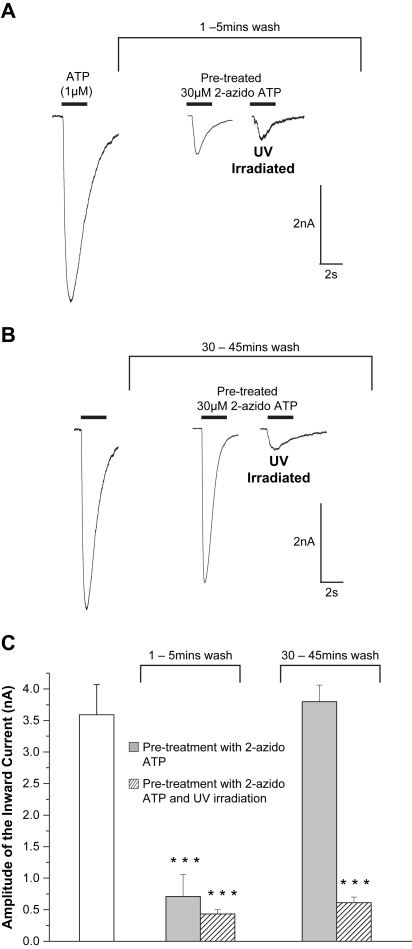
Effect of cross-linking 2-azido ATP to the P2X1 receptor. HEK293 cells which stably expressed the P2X1 receptor were pre-treated in either 30 μM 2-azido ATP and UV irradiated for 3 min or 30 μM 2-azido ATP (without UV irradiation). They were then placed on the electrophysiology rig and were continually washed in extracellular solution for the time described. ATP (1μM) was then applied (application indicated by black bar). (A) Whole cell patch clamp traces recorded 1–5 min after washout of 2-azido ATP (for representative cells) show the reduction in ATP response caused by desensitisation of the P2X1 receptor. (B) Traces show the recovery of ATP evoked currents in cells pre-treated with 2-azido ATP (without UV). The inhibited response of cells pre-treated in 2-azido ATP and UV irradiated suggests cross-linking. (C) Pooled data shows the effects of each scenario (*p < 0.05, **p < 0.01 and ***p < 0.001 show significant difference from the control). Error bars indicate SEM.

To test whether 2-azido ATP could cross-link to the receptors, cells were treated in 30 μM 2-azido ATP (a maximal agonist concentration) and UV irradiated for 3 min. The cells were then placed in the electrophysiology recording chamber and perfused. Whole cell patch clamp recordings were made from cells over a 45 min period. Results showed that the response produced by 1 μM ATP application was reduced by 86% to −0.49 ± 0.09 nA (*n* = 30) a decrease from −3.47 ± 0.9 nA (*n* = 9) which was recorded from cells which had not been UV irradiated (Fig. 2). This reduction in ATP responsiveness suggested cross-linking of the compound to the receptor binding site. As a control, cells were pre-treated in 30 μM 2-azido ATP (without UV irradiation) and the responses were recorded over a 45 min time period. Within 1–5 min the responses were significantly reduced to −0.71 ± 0.35 nA (*n* = 10). However, this reduction results most likely from the time required for P2X1 receptors to recover from desensitisation as the peak amplitude responses recover after continual wash with extracellular solution. Within 30–45 min the responses from cells pre-treated in 30 μM 2-azido ATP increased to −3.8 ± 0.26 nA (*n* = 10). These results suggest that the reduction in response brought about by the UV irradiation of cells in the presence of 2-azido ATP was caused by the cross-linking of 2-azido ATP within the P2X1 ATP binding domain.

### 3.4 Receptor protection with ATP or suramin

**Fig. 3:**
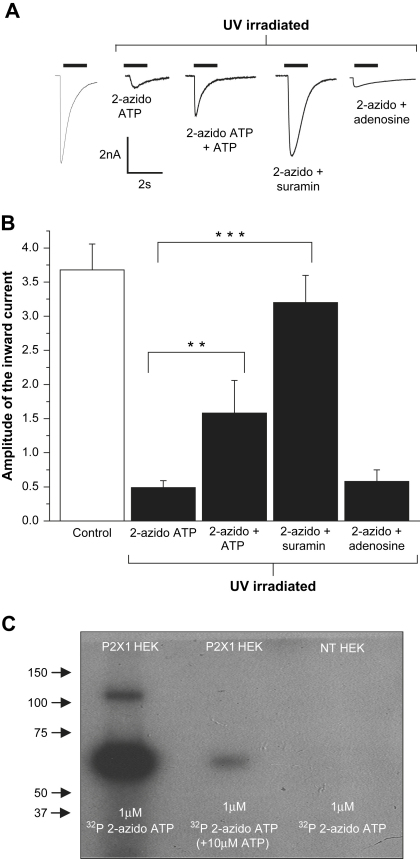
The cross-linking effects of 2-azido ATP reduced by receptor protection with excess ATP or suramin. (A) Representative currents evoked by ATP (1 μM) evoked from HEK293 cells stably expressing the P2X1 receptor under control conditions, following 2-azido ATP and irradiation or pre-treated 300 μM ATP, 100 μM suramin or 300 μM adenosine in addition to 30 μM 2-azido ATP and UV irradiated for 3 min. Patch clamp traces show the reduction in inhibition by 2-azido ATP and UV irradiation caused by protection of the P2X1 receptors by ATP or suramin. The addition of adenosine did not protect the P2X1 receptors from the inhibitory effect of 2-azido ATP. (B) Summary data from A of ATP (1 μM) evoked responses (**p < 0.01 and ***p < 0.001). Error bars indicate SEM. (C) Samples of non-transfected HEK293 cells and HEK293 cells stably expressing the P2X1 receptor were treated with 1 μM (0.32 MBq) 2-azido-[γ^32^P] ATP. In addition, cells stably expressing the P2X1 receptor were treated with 10 μM ATP and 1 μM 2-azido ATP. All samples were UV irradiated for 3 min. After lysis, samples were immunoprecipitated with the P2X1 antibody (Alomone) and ran on a 10% SDS-PAGE gel. After drying, the gel was exposed to autoradiography film for 3 days at −80 °C. Intense bands appeared at a similar weight range for the glycosylated monomer (∼55 kDa) and dimer (∼110 kDa) of P2X1 protein and suggest successful labelling by 2-azido ATP. 2-Azido treatment (1 μM) shows intense binding to the P2X1 receptor but, as indicated in the electrophysiological study, this binding was reduced when the cells were treated with ATP (10 μM).

To further test 2-azido ATP's validity as a cross-linking compound at the P2X1 receptor, receptor protection experiments were conducted. The P2 receptor antagonist suramin (100 μM), or a supramaximal concentration of ATP (300 μM), was used to pre-treat the cells and then they were UV irradiated in the presence of either ATP or suramin along with 30 μM 2-azido ATP. Cross-linking by 2-azido ATP caused the subsequent peak current responses from P2X1 cells to be reduced by 86% (see above). P2X1 receptors that were treated in 300 μM ATP, prior to UV irradiation in the presence of 30 μM 2-azido ATP were reduced, compared to controls, by 57% to −1.58 ± 0.48 nA (*n* = 12); a significantly smaller reduction then when treated with 2-azido ATP irradiation alone (*p* < 0.01). P2X1 receptors that were treated with 100 μM suramin, prior to and during UV irradiation in the presence of 30 μM 2-azido were protected from inhibition (*p* < 0.001) and ATP P2X1 receptor currents were only reduced by 13% to −3.2 ± 0.42 nA compared to controls (*n* = 23) (Fig. 3).

One possibility for the protection of the current response is that the addition of ATP or suramin caused an increased absorbance of UV light. This is called molar extinction. Molar extinction refers to the potential absorbance of the UV light by the molecules of ATP or suramin. If the light was absorbed by these molecules, then less light would have been able to penetrate down to the P2X1 receptors themselves. To test this theory, 300 μM adenosine was used. Adenosine has no pharmacological action at the P2X receptors and so should not provide protection from the effects of UV irradiated 2-azido ATP. The level of inhibition following 2-azido ATP irradiation was the same whether adenosine (300 μM) was present or absent (reduction to −0.53 ± 0.17 nA (*n* = 20, Fig. 3)). This shows that the reduction in the effects of 2-azido ATP by suramin or ATP do not result from a molar extinction effect and demonstrates that 2-azido ATP is acting at the ATP binding site on the P2X1 receptor and could be used to label the P2X1 receptor. One consideration is that reactive 2-azido ATP, and the other reactive analogues (see later), may diffuse away from the ATP binding site following UV irradiation and could react with nearby residues. However, in studies on the Na,K-ATPase where 2-azido was used in a similar way following peptide sequencing it was shown to cross-link to a single residue within the ATP binding site (Tran et al., 1994) suggesting that diffusion of the reactive compound away from the binding site is not a major contaminating factor.

### 3.5 Radiolabelling of P2X1 receptors with 2-azido [γ^32^P] ATP

The cross-linking of 2-azido ATP and the P2X1 receptor was tested using a radiolabelling assay. HEK293 cells stably expressing the P2X1 receptor were treated with 1 μM (0.32 MBq) 2-azido [γ^32^P] ATP. To determine the level of non-specific binding, non-transfected HEK293 cells were treated similarly. Both sets of cells were UV irradiated and the cells were homogenised. To reduce non-specific binding the cross-linked P2X1 receptor was isolated by immunoprecipitation with the P2X1 antibody and the samples were run on a 10% SDS gel. Exposure of the dried gel to autoradiography film produced intense bands which corresponded to the sizes of the glycosylated P2X1 monomer (∼55 kDa) and a small amount of dimer (∼110 kDa). The intensity of these bands was markedly reduced when P2X1 cells were pre-treated with 10 μM ATP (in addition to 2-azido ATP). This indicated that ATP protected the receptors from cross-linking to 2-azido ATP by occupying the majority of sites prior to UV irradiation (Fig. 3C) and suggests that the majority of the photo-reactive compound does not bind randomly to the P2X1 receptor just because of close proximity of the compound at the time of irradiation. In conclusion this evidence corroborates the findings made earlier and directly suggests that ATP and 2-azido ATP compete for the same binding site on the P2X1 receptor. It confirms the identification of 2-azido ATP as a cross-linking compound which directly binds to the P2X1 receptor.

### 3.6 The effects of 2-azido ATP at other P2X receptor subtypes

**Fig. 4:**
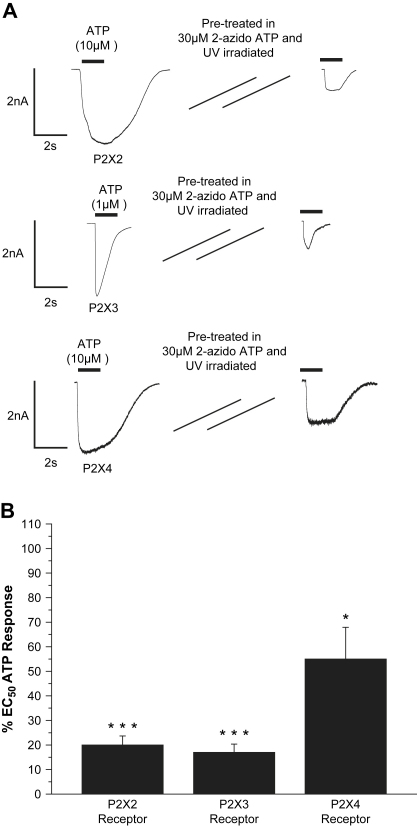
Cross-linking effect of 2-azido ATP at other P2X subtypes. HEK293 cells stably expressing the human P2X4 receptor and cells transfected with rat P2X2 and rat P2X3 DNA were subjected to similar experiments as those conducted at the P2X1 receptor. As before, cells were treated in 30 μM 2-azido ATP and UV irradiated for 3 min and tested with their respective EC_50_ concentration of ATP. (A) Whole cell patch clamp traces show representative currents to ATP at EC_50_ concentration for different P2X subtypes under control conditions and following UV irradiation in the presence of 2-azido ATP. (C) Summary data shows the inhibition of responses to an EC_50_ concentration of ATP by UV irradiation with 2-azido ATP (*p < 0.05 and ***p < 0.001). Error bars indicate SEM.

Previous studies have shown that there are differences between the pharmacology of the various subtypes of the P2X receptor. The ability of 2-azido ATP to cross-link to particular P2X subtypes may reflect differences in their respective ATP binding domains. In the presence of 2-azido ATP, HEK293 cells expressing P2X2–P2X4 were all UV irradiated for 3 min. Subsequent patch clamp recordings monitored changes in the responses to their approximate EC_50_ ATP concentrations (Fig. 4). ATP (1 μM) was used for P2X3 expressing cells and 10 μM ATP was used for P2X2 and P2X4 cells. ATP (at EC_50_ concentration of ATP) was applied to untreated P2X2–P2X4 cells as a control.

At the P2X2 receptor 10 μM ATP application evoked inward currents that slowly desensitised. The mean peak current was −2.3 ± 0.45 nA (*n* = 5). Irradiation of these cells in the presence of 2-azido ATP caused the mean peak current to reduce to −0.46 ± 0.07 nA (*n* = 18). This was an 80% reduction in the current response (*p* < 0.001). The application of 1 μM ATP at P2X3 receptors caused transient inward currents that quickly desensitised. The mean peak current was −2.12 ± 0.38 nA (*n* = 5). At P2X3 receptors the mean peak current was reduced to −0.35 ± 0.09 nA (*n* = 12), a reduction of 83% (*p* < 0.001). The application of 10 μM ATP to P2X4 receptors evoked transient inward currents that were slow to desensitize. The mean peak current was −1.06 ± 0.24 nA (*n* = 5). The peak amplitude of the response was reduced to −0.58 ± 0.13 nA (*n* = 9). This was a 45% reduction (*p* < 0.05). These results suggest that 2-azido ATP can be used to cross-link a range of P2X receptors.

### 3.7 Effects of ATP azidoanilide at P2X1 receptors

**Fig. 5:**
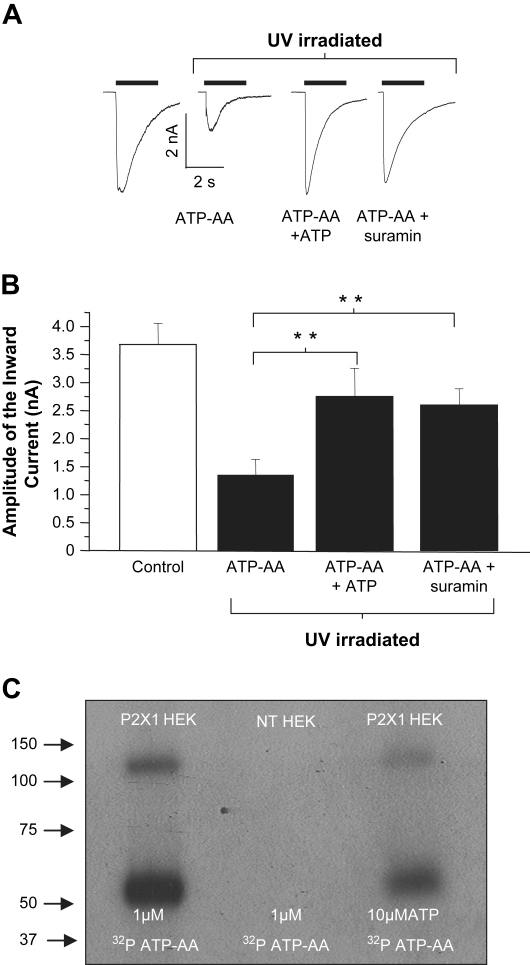
Effects of ATP azidoanilide on P2X1 receptors. (A) Representative currents evoked by ATP (1 μM) evoked from HEK293 cells stably expressing the P2X1 receptor under control conditions, following ATP azidoanilide (ATP-AA, 100 μM) and irradiation or pre-treated 300 μM ATP, 100 μM suramin in addition to 100 μM ATP-AA and UV irradiated for 3 min. Patch clamp traces show the reduction in inhibition by ATP-AA and UV irradiation caused by protection of the P2X1 receptors by ATP or suramin. (B) Summary data from A (**p < 0.01 and ***p < 0.001). Error bars indicate SEM. (C) Radiolabelled binding studies with P2X1 protein. Non-transfected HEK293 (NT HEK) cells and HEK293 cells stably expressing the P2X1 receptor were pre-treated in 1 μM (0.92 MBq) ATP azidoanilide-[γ^32^P]. In addition, cells stably expressing the P2X1 receptor were treated with 10 μM ATP and 1 μM azidoanilide. All samples were UV irradiated for 3 min. After lysis, the protein was immunoprecipitated with the P2X1 antibody (Alomone) and ran on 10% SDS-PAGE. After drying, the gel was exposed to autoradiography film for 4 days at −80 °C. Intense bands appear at a similar weight range for the glycosylated monomer (∼55 kDa) and dimer (∼110 kDa) for the P2X1 protein and suggest successful labelling by ATP azidoanilide. ATP azidoanilide (1 μM) treatment shows strong binding to the P2X1 receptor this binding was reduced when ATP (10 μM) was co-applied.

**Fig. 6:**
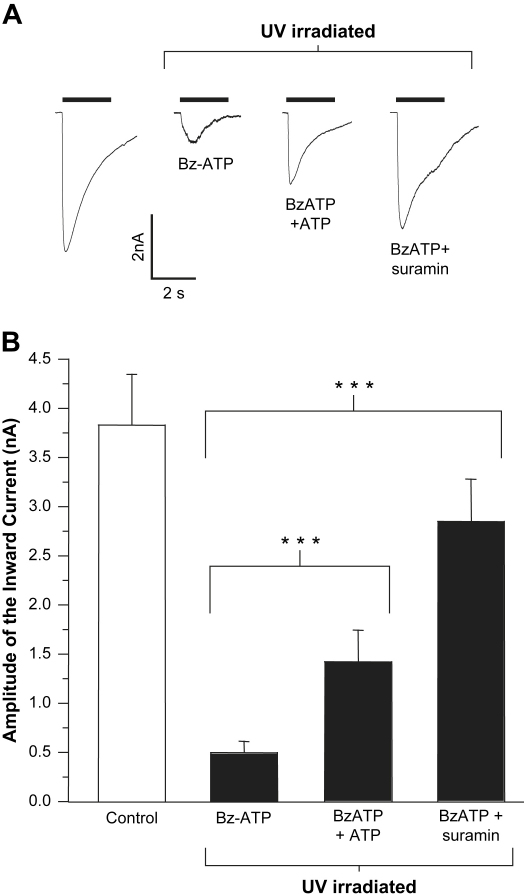
Effects of UV irradiation of BzATP on P2X1 receptor currents and protection by ATP and suramin treatment. (A) Representative ATP (1 μM, application indicated by bar) evoked currents from P2X1 receptors stably expressed in HEK293 cells from untreated control cells (left panel) or following 5 min incubation with BzATP (10 μM) followed by 3 min UV irradiation and a subsequent 5 min washout period to allow receptors to recover from desensitisation, or co-application of BzATP and ATP or BzATP and suramin followed by 5 min washout. (B) Summary bar graph shows that pre-treating cells with either 300 μM ATP or 100 μM suramin reduced current inhibition by BzATP (***p < 0.001 from UV irradiation with 10 μM BzATP alone).

ATP azidoanilide (ATP-AA) has a substituted reactive azidoanilide moiety at the end of the gamma phosphate of ATP. ATP-AA (1 μM) was ineffective as an agonist at P2X1 receptors, however, responses were evoked at 100 μM (mean amplitude −2.1 ± 0.4 nA, *n* = 10). When cells expressing the P2X1 receptor were treated ATP-AA (100 μM) and UV irradiated currents evoked by ATP (1 μM) were reduced from a control in the absence of ATP-AA of 3.7 ± 0.7 nA (*n* = 10) by 67% to −1.2 ± 0.4 nA (*n* = 30) (*p* < 0.01). This reduction in P2X1 receptor current by UV treatment with ATP-AA was reduced significantly (*p* < 0.01 for each) to 25 and 30% by co-application of ATP (300 μM) or suramin (100 μM), respectively (Fig. 6). These results suggest that ATP-AA directly cross-links to the ATP binding site of the P2X1 receptor following UV irradiation. To test this directly we determined whether radiolabelled ATP-AA could be cross-linked to P2X1 receptors. Following ATP-AA-[γ^32^P](1 μM) treatment radioactivity associated with an ∼55 kDa protein was recorded from HEK293 cells expressing the P2X1 receptor but not non-transfected HEK293 control cells. The P2X1 receptor specific labelling was reduced by >90% by co-application with 10 μM ATP at the time of irradiation (Fig. 5). Taken together these results demonstrate that ATP-AA can be used to cross-link to P2X1 receptors.

### 3.8 Effects of BzATP on P2X1 receptor currents

BzATP is an ATP analogue with substitutions on the ribose and has been used to label the nucleotide binding site of P2Y receptors (Erb et al., 1993). BzATP (10 μM, a maximal concentration; Roberts and Evans, 2004) evoked P2X1 receptor currents (−4.5 ± 0.7 nA, *n* = 14) of equivalent amplitude to an EC_50_ concentration of ATP (1 μM, peak amplitude 3.7 ± 0.4 nA, *n* = 5) consistent with its action as a partial agonist at the P2X1 receptor (Roberts and Evans, 2004). ATP (1 μM) evoked responses were reduced by 89% (to −0.5 ± 0.1 nA, *n* = 52) following incubation with BzATP (10 μM) and UV irradiation (Fig. 6). BzATP treatment alone had no effect on ATP evoked currents indicating that the reduction following UV irradiation results from BzATP cross-linking to the receptor locking the receptor in a desensitised state. The inhibition by UV irradiation in the presence of BzATP could be significantly reduced by co-application with either ATP (300 μM) or suramin (100 μM) to 61 and 23%, respectively (Fig. 6). These results suggest that the BzATP is acting to cross-link to the P2X1 receptor at the ATP binding site.

## 4 Discussion

**Fig. 7:**
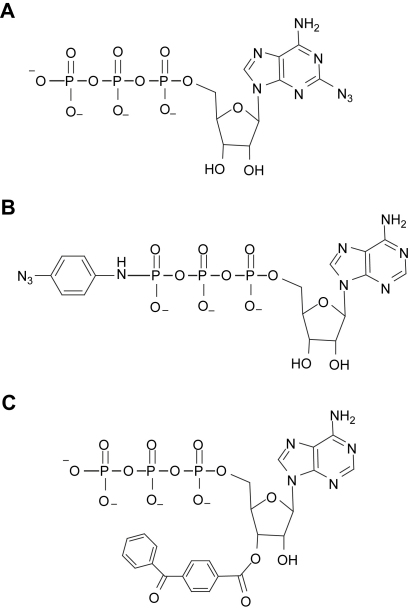
Structures of reactive ATP analogues. (A) 2-Azido ATP. (B) ATP azidoanilide. (C) 2′,3′-*O*-(4-benzoylbenzoyl)-ATP.

P2X receptors comprise a distinct family of ligand gated ion channels and sequence analysis suggests that they have a novel mechanism of ATP binding. In the absence of a solved structure for the P2X receptor site-directed mutagenesis studies have been used to identify regions of the receptor that are important for determining ATP actions. Cross-linking of nucleotide analogues to a variety of proteins has been useful in determining the site of ATP binding. In this study we have shown that the photo-reactive compounds 2-azio ATP, ATP-AA and BzATP (for structures see Fig. 7) are agonists at the receptor and useful for the study of agonist binding at P2X receptors. The reactive side groups of these substituted ATP analogues cross-link to the P2X receptor at sites that are likely to be close to those involved in ATP binding.

FSBA has adenosine and ribose moieties but does not have a phosphate tail. In place of the first phosphate group there is a carbonyl group, the second phosphate is replaced by a benzene ring and the terminal phosphate is replaces by a sulfonyl fluoride moiety. The reactive sulfonyl fluoride group confers the ability of FSBA to bind to a broad range of nucleotide binding sites. It has a reactive sulfonyl fluoride group that is an electrophilic agent in covalent reactions with several types of amino acids; these include tyrosine, lysine, histidine, serine and cysteine (Colman, 1983). Nucleotide binding sites as diverse as the ADP receptor on platelets (Figures et al., 1987), the binding domain on the ArsA protein of *E. coli* (Ramaswamy and Kaur, 1998) and the nucleotide binding site on the enzyme pyruvate kinase (Wyatt and Colman, 1977) have been successfully labelled with FSBA. However, in the present study FSBA was ineffective as an agonist or cross-linker at P2X1 receptors. The lack of agonist action of FSBA and inability to block subsequent ATP evoked responses shows that FSBA does not bind to the receptor and is consistent with the lack of effects of ADP as an agonist or antagonist at the receptor and supports the concept that the P2X receptor drugs need to have adenine, ribose and triphosphate groups to be reactive. The lack of effect of FSBA also suggests once again that the ATP binding site of P2X receptors is different from those of many other ATP binding proteins.

2-Azido ATP was effective as an agonist at the P2X1 receptor and this is consistent with previous reports showing that 2-chloro and 2-methyl thio ATP (Evans et al., 1995) are agonists at the P2X1 receptor and suggest that substitutions at the 2nd position on the adenine ring are well tolerated. The binding of 2-azido ATP (and for ATP-AA and BzATP) leads to activation and desensitisation of the receptor. Thus it seems likely that following UV irradiation the 2-azido ATP (and ATP-AA and BzATP) inhibits subsequent ATP responses by locking the receptor in an agonist bound desensitised state. This also demonstrates that the agonist needs to unbind from the receptor in order for recovery from desensitisation. The blocking of the inhibitory actions of 2-azido ATP and UV irradiation by application of ATP and suramin, as well as the radioligand binding experiments supports that 2-azido ATP mediates its effects following UV irradiation by acting at the ATP binding site. We have used radiolabelled 2-azido ATP to study changes in agonist sensitivity at P2X1 receptor mutants (Roberts and Evans, 2007). The success of cross-linking 2-azido ATP was not restricted to the P2X1 receptor; ATP responses at P2X2–P2X4 receptors were also significantly inhibited when they were UV irradiated in the presence of 2-azido ATP. This suggests that such a radiolabelling assay may also be useful for these other P2X receptor subtypes.

ATP-AA was an agonist at the P2X1 receptor, and following UV irradiation could be cross-linked to the receptor agonist binding site as assessed by the reduction of subsequent ATP responses or the incorporation of the radiolabelled agonist. This shows that substitutions at the end of the phosphate tail are well tolerated as has been shown in previous studies using for example diadenosine pentaphosphate (for review see Evans, in press). This suggests that the agonist binding pocket for the terminal phosphate is somewhat open-ended so that it can tolerate/incorporate the substitutions.

BzATP is a partial agonist at the P2X1 receptor indicating that the binding energy associated with ligand recognition is not sufficient to fully activate the receptor. The substitution of the benzoyl group off the ribose suggests that this part of the ATP molecule does not form close contact with the receptor, indicating that this part of the molecule may be on the periphery of the ATP binding site. The ability of BzATP, following UV irradiation, to block subsequent ATP evoked responses demonstrates that the benzoyl group can form close contact and cross-link to the P2X1 receptor.

In summary we have shown that photo-reactive ATP analogues with substitutions on the adenine ring, ribose and phosphate tail can be cross-linked to the P2X1 receptor. This may prove useful for providing direct evidence for the local environment of ATP binding at the family of P2X receptors.
